# Supplementary material for: Identification of Prognostic Markers of Gynecologic Cancers Utilizing Patient-Derived Xenograft Mouse Models
Source: Cancers (Basel). 2022 Feb 6;14(3):829. doi: 10.3390/cancers14030829 (PMC8834149; doi:10.3390/cancers14030829)

Supplementary Figure S1

PDX tumors maintain the histology of their corresponding original tumors. Representative image of hematoxylin and eosin (H&E) staining of tumors from ovarian cancer, cervical cancer, and uterine cancer (x200).

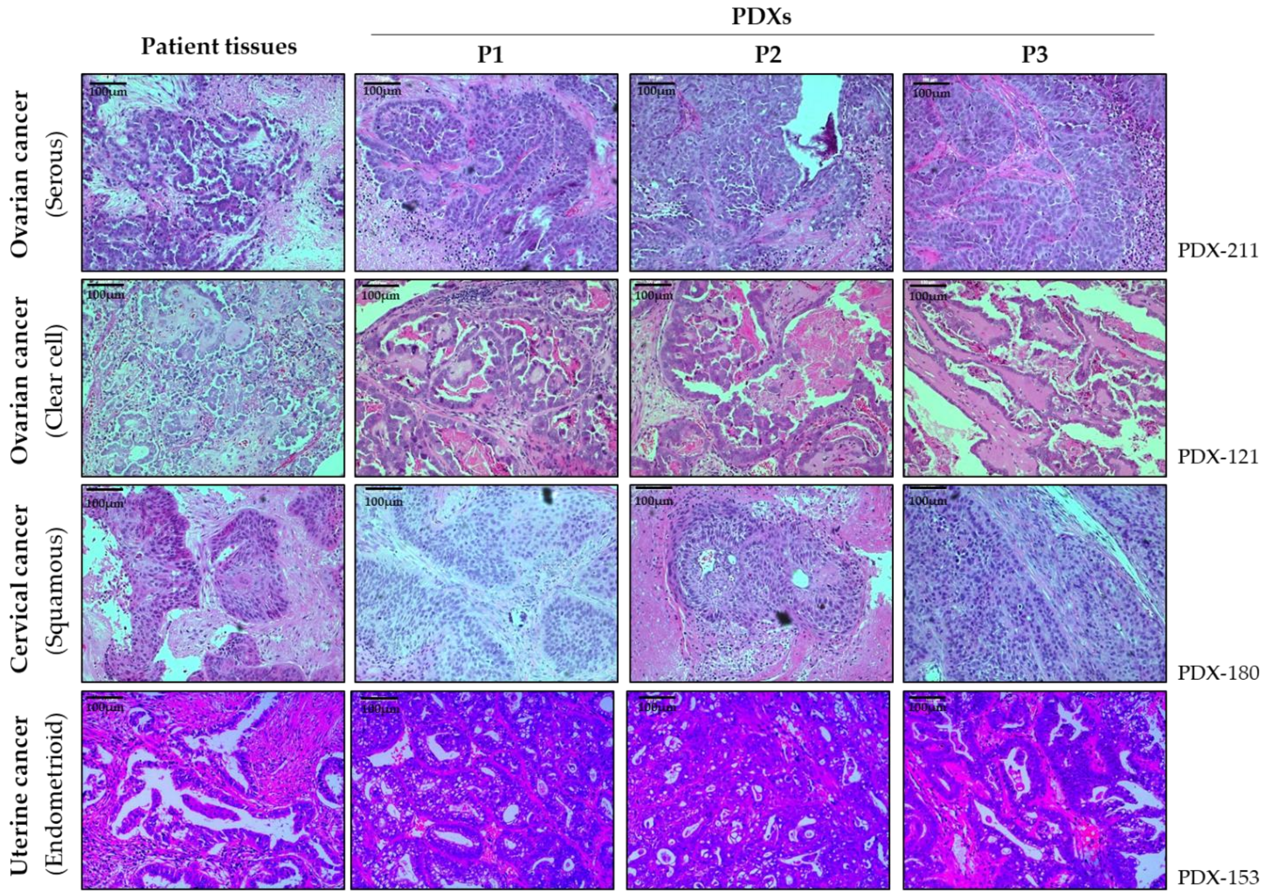

Supplementary Figure S2

Overall survival of patients based on P1 tumor engraftment status with epithelial ovarian cancer (A), stage III epithelial ovarian cancer (B). Overall survival of patients based on P1 tumor growth rate with epithelial ovarian cancer (C), epithelial ovarian cancer stage III. Kaplan–Meier survival analysis was carried out, and log–rank p–values and a Gehan–Breslow–Wilcoxon test, HR, and CI are shown for each of the results. HR: hazard ratio, CI: confidence interval

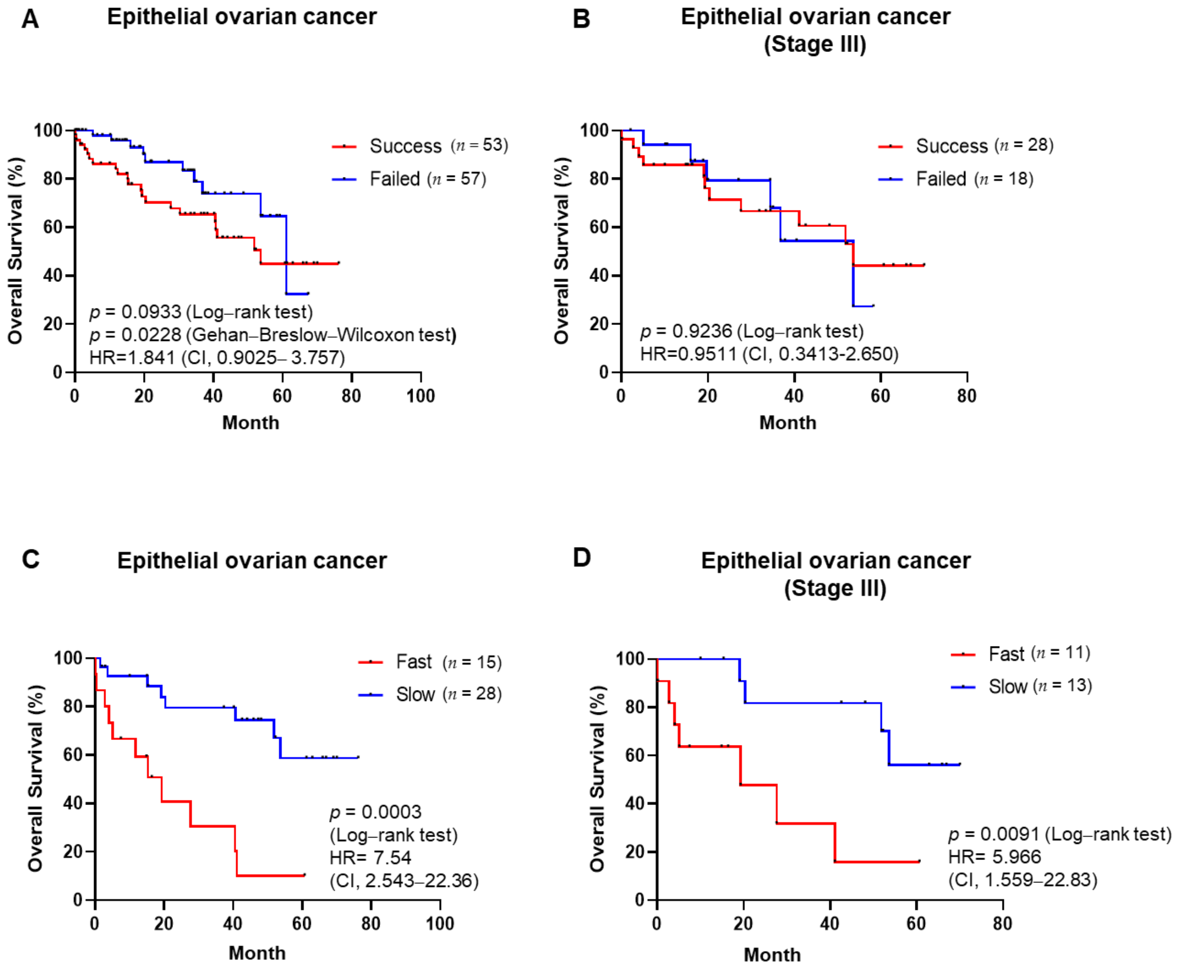

Supplement: Supplementary file 1 [file cancers-14-00829-s001.zip › cancers-1508809-supplementary.pdf]
